# Structural Regulation and Electroconductivity Change of Nitrogen-Doping Reduced Graphene Oxide Prepared Using *p*-Phenylene Diamine as Modifier

**DOI:** 10.3390/nano7100292

**Published:** 2017-09-25

**Authors:** Tiefeng Peng, Hongjuan Sun, Tongjiang Peng, Bo Liu, Xiaolong Zhao

**Affiliations:** 1 Key Laboratory of Ministry of Education for Solid Waste Treatment and Resource Recycle, Southwest University of Science and Technology, Mianyang 621010, China; pengtiefeng@cqu.edu.cn (T.P.); ptf1242@swust.edu.cn (T.P.); liubs1234@163.com (B.L.); zhaoxi1010@163.com (X.Z.); 2 Institute of Mineral Materials and Applications, Southwest University of Science and Technology, Mianyang 621010, China

**Keywords:** nitrogen doping, regulation, conjugation

## Abstract

Using *p*-phenylene diamine (PPD) as a modifier and nitrogen resource, nitrogen-doping reduced graphene oxide was prepared by one-step refluxing method. The influence of PPD-GO (graphene oxide) mass ratio *X* on surface functional groups, layer structure, and electroconductivity of nitrogen-doping reduced grapheme oxide (NRGO-*X*) was investigated by Fourier Transform Infrared Spectroscopy (FT-IR), X-ray photoelectron spectroscopy (XPS), X-ray diffraction (XRD), UV-vis absorption spectrum, and electrical measurement. The results showed that GO can be simultaneously reduced and nitrogen-doped by PPD. When PPD-GO mass ratio *X* ≤ 6, there existed three types of N configurations in NRGO-X, including pyridinic N, pyrrolic N, and graphitic N. However, when *X* > 6, the pyridinic N disappeared in a six-membered ring. Further, the reduction process of NRGO as well as the nitrogen doping level and type can be regulated by changing the mass ratio *X*. With the increase of *X*, the *d*-spacing of NRGO-X layers increased first and then decreased, while the electrical conductivity increased gradually.

## 1. Introduction

With high conductivity [1] and thermal conductivity [2], as well as good flexibility and mechanical properties [3], graphene [4,5] or graphene oxide (GO) [6,7] has attracted wide attention from researchers in chemistry, physics, and materials fields [8,9,10] since its discovery in 2004. In order to eliminate the influence of the gapless and inert sp^2^ hybrid carbon atom on GO [11,12], researchers have made attempts to regulate the structure and properties of graphene oxide by substituting the carbon atom with a doping nitrogen atom (N).

N doping can effectively change the spin density and charge distribution of adjacent carbon atoms, and induce more positive charges to move to the adjacent carbon atoms, so as to improve the performance of anion exchange [13,14]. In addition, N-doped graphene oxide has a wide range of potential applications [15] in super capacitors, lithium batteries, electrochemical sensors, and fuel cells. Particularly in the oxygen reduction reaction (ORR), N-doped GO has excellent electrocatalytic activity. Therefore, it is expected to replace expensive Pt-based catalysts [14] in fuel batteries.

There are currently many methods for the preparation of N-doped GO [15,16]; among them, the chemical vapor deposition (CVD) method has advantages in obtaining multiple N configuration types [17,18,19]. Luo et al. [20] prepared single pyridine N-doped GO in the presence of ammonia by introducing hydrogen and ethylene through CVD on copper foil. By adjusting the flow rate of ammonia, the N-C atomic ratio can be adjusted from 0% to 16%. The results showed that the pyridine N can effectively change the valence band structure of GO, by increasing π electron density near the Fermi level and reducing the performance function. However, unlike simply treating GO in N-containing precursors, the CVD method is more complex and the size of the product is restricted. As the precursor for large-scale preparation of graphene, GO features a low price and contains a large number of oxygen-containing functional groups. The existence of these oxygen-containing functional groups makes it have higher reactivity [21,22], and allows it to introduce the N atom or N-containing active functional groups into the GO; therefore, it can be used as initial material for the preparation of N-doped GO.

At present, N-containing organic small molecules, such as NH_3_/N_2_H_4_ [23], urea CON_2_H_4_ [24], Dicyandiamide C_2_H_4_N_4_ [25], as well as GO, have been used as precursors to prepare N-doped GO. Sun et al. [26] prepared N-doped GO by heating the mixed solution of GO and ammonium nitrate at 350 °C for 1 h using ammonium nitrate as the nitrogen doping and reducing reagent. However, the types and levels of incorporated N atoms cannot be well controlled. Therefore, in order to better understand the influence of the structural change on product performance, it is of great importance to regulate the type and level of doped N atoms. Moreover, the relationship between the structure and properties of NRGO-*X* with different types and levels of N-doping will also be crucial.

Owing to its special conjugated structure with a twinning-amino group, PPD can react with the oxygen-containing functional groups of GO. It can achieve functional modification, as well as reduction, N-doping, and cross-linking at the same time. In this study, NRGO-*X*s with different N-doping and reduction degrees were prepared by the one-step hydrothermal refluxing method using PPD (conjugated N-containing aromatic amine) as a nitrogen source and modifier, by changing the mass ratio of PPD and GO. Via FT-IR, XPS, XRD, UV-vis, and other testing methods, the surface functional groups, structural properties, and electrical conductivity change of prepared NRGO-*X* were investigated. Thus, the influence of the increase of *X*, the change of surface oxygen functional groups, the change of N doping level, and type on the layer structure and electrical conductivity were revealed.

## 2. Experimental Section

### 2.1. Raw Materials and Reagents

Natural flake graphite (Tangseng ditch, Xinghe County, Inner Mongolia, with a carbon content above 90%, −200 mesh), Potassium Permanganate (KMnO_4_), concentrated sulfuric acid (98%, H_2_SO_4_), hydrogen peroxide solution (5%, H_2_O_2_), and hydrochloric acid solution (mol·L^−1^, HCl) were purchased from Chengdu Jinshan Chemical Reagent Co., Ltd., Chengdu, China; *p*-Phenylene Diamine (PPD), methanol (99.5%, CH_3_OH) were purchased from Chengdu Kelong Chemical Reagent Factory. All reagents used were analytical grade, and the water used throughout experiments was deionized water.

### 2.2. Sample Preparation

GO was synthesized using natural graphite powder by the improved Hummers method. The preparation of nitrogen-doped reduced graphene oxide (NRGO-X) was as follows. Firstly, 0.2 g of graphite oxide powder was put into 250 mL deionized water for 120 min of ultrasonic dispersion, resulting in GO dispersion of 0.8 mg/mL; secondly, 0.4 g PPD was added into the GO dispersion and ultrasonic mixing was conducted for 10 min; then, the mixed solution was put into a 500-mL three-neck flask, under water bath magnetic stirring and reflux at 90 °C for 24 h. Subsequently, the mixed solution was filtered by polypropylene (PP) thin film with the average pore diameter of 0.2 μm, and the polypropylene thin film was washed with ethanol and deionized water five times.

**Figure 1:**
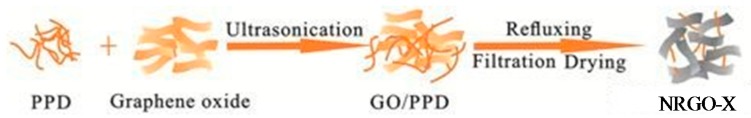
The preparation process of the PPD nitrogen-doped reduced graphene oxide.

The preparation process is shown in Figure 1. Finally, the thin film was dried at 80 °C for 24 h, and the desired samples were obtained. The above operations were repeated, changing the doping amount of PPD to 0.8 g, 1.2 g, 1.6 g, and 2 g, in order to obtain N-doped reduced graphene oxide samples with different PPD/GO mass ratios, which were marked as NRGO-X (*X* = 2, 4, 6, 8, 10. *X* represents PPD/GO mass ratio).

### 2.3. Sample Characterization

X-ray diffraction analysis (XRD) was performed by an X’ pert MPD Pro type X-ray diffractometer (PANalytical B.V., Almelo, Holland), Cu target, DS: (1/2)°, SS: 0.04 rad, AAS: 5.5 mm. FT-IR was performed by a Nicolet-5700 type infrared spectrometer (Thermo Nicolet Corporation, Madison, WI, USA) with a scanning range of 4000~500 cm^−1^, wherein the samples were prepared by the KBr compression method. Ultraviolet visible (UV-vis) analysis and tests were conducted by a UV-3150 ultraviolet visible near infrared spectrometer (Shimadzu Corporation, Kyoto, Japan) with ultra-pure water as the reference within a test range of 200~500 nm.

XPS was carried out by an XSAM800 type multifunctional surface analysis electron spectrometer (Kratos, Manchester, UK) with Al target (1486.6 eV), X light gun power of 12 KV × 15 mA by the fixed analyzer transmission (FAT) method. The data was corrected by pollution carbon C1s (284.8 eV). Resistivity test was conducted by an SZT-2A type four-probe tester (Suzhou Tongchuang Electronics Co., Ltd., Suzhou, China) and KDY-1 type four-probe resistivity-resistance tester (Guangzhou Kunde Science and Technology, Co., Ltd., Guangzhou, China). Peak-fit processing of XPS peaks was carried out by Casa XPS software (version 2.3.14, Casa Software Ltd., Devon, UK), wherein the deduction of background was completed by the Shirley method, and the fitting function was the Gaussian-Lorentzian composite function.

## 3. Results and Discussion

### 3.1. Change of Surface Functional Groups before and after Functionalization

**Figure 2:**
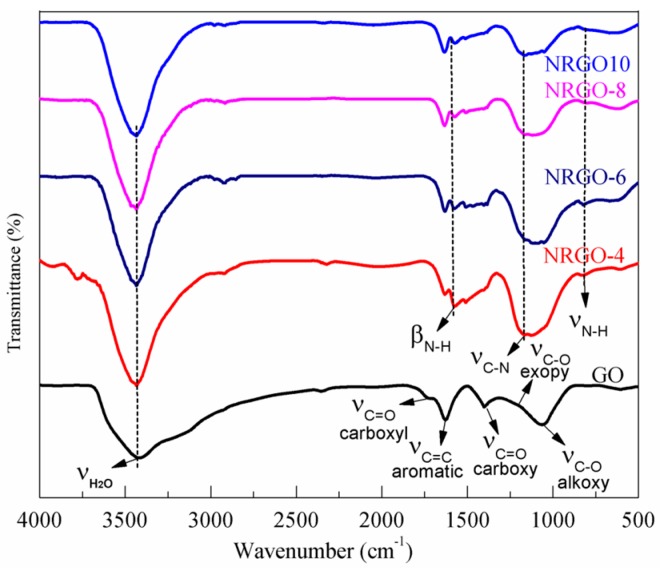
FT-IR spectra of GO and NRGO-X (*X* = 4, 6, 8, 10).

Figure 2 shows the FT-IR of GO and NRGO-*X*. It can be seen that many oxygen functional groups exist on the GO structure. The absorption peaks at 3423, 1713, 1631, 1396, 1186, and 1060 cm^−1^ are ascribed to the stretching vibration peaks of water molecules (H_2_O), carbonyl (C=O), aromatic skeleton carbon ring (C=C), carboxyl (O–C=O), epoxy (O–C–O), and alkoxy (C–O), respectively [27,28]. Three new absorption peaks observed at 820, 1585, and 1175 cm^−1^ are attributed to the stretching vibration of N–H, the bending vibration of N–H, and the stretching vibration of C–N, respectively [29,30,31].

Compared with GO, the absorption peaks of O–C=O and C–O–C in NRGO-X have lowered intensity and decreased content, which is due to the consumption of oxygen (O) caused by the reaction between PPD and oxygen functional groups. Moreover, the electron-withdrawing aromatic ring structure of PPD enhances the reduction of GO oxygen-containing groups.

**Figure 3:**
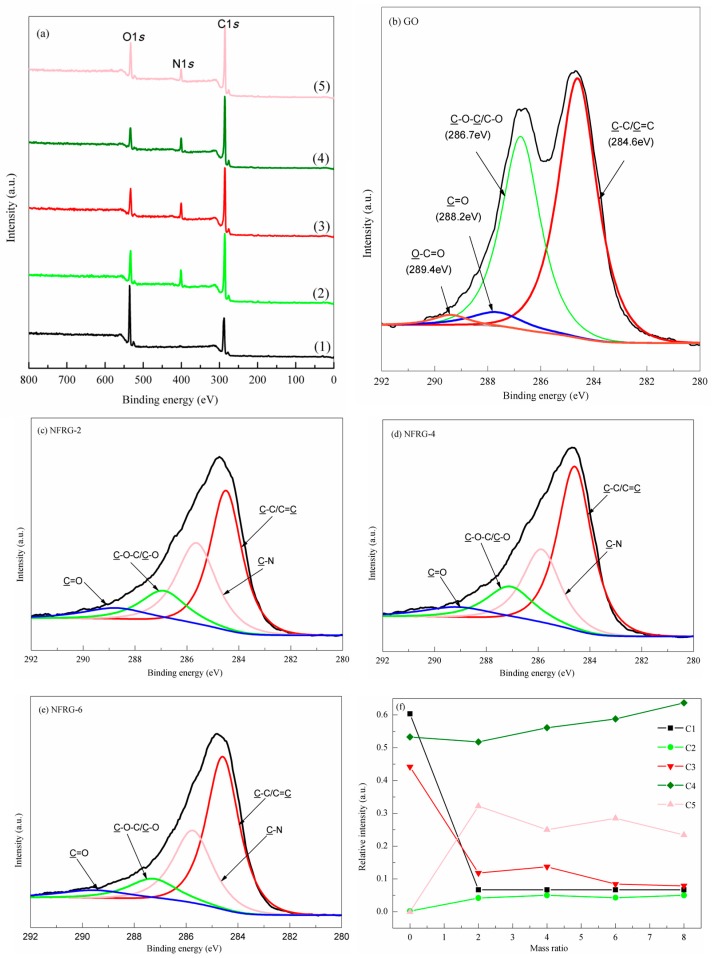
(**a**) XPS data of GO and NRGO-X (*X* = 2, 4, 6, 8), (1) GO, (2) NRGO-2, (3) NRGO-4, (4) NRGO-6, (5) NRGO-8. (**b**–**e**) C1*s* spectra of GO and NRGO-X (*X* = 2, 4, 6). (**f**) Relative intensity of different C species with the *X*.

To further identify the change of functional groups, XPS full spectrum analyses of GO and NRGO-X (*X* = 2, 4, 6, 8) were carried out, as shown in Figure 3a. It can be seen that GO does not contain N, but only displays two characteristic peaks at 289 eV and 535 eV, which are ascribed to the C1s and O1s spectra [32], respectively. In contrast, NRGO-X shows a new N1s spectrum at 400 eV, which indicates the existence of N atoms.

Based on the above analyses, peak-differentiation-imitating of XPS C1s peaks of GO and NRGO-*X* was conducted, as shown in Figure 3b–e. According to Figure 3b, the XPS C1s spectrum of GO consists of four peaks at 289.4, 288.2, 286.7, and 284.6 eV, which are attributed to the characteristic peaks of O–C=O (labeled as C1), C=O (C2), C–O–C/C–O (C3), and C–C/C=C (C4) [31], respectively. In addition, as shown in Figure 3c–e, a new characteristic peak of NRGO-*X* appears near 285.6 eV, which can be attributed to C–N (labeled as C5) [29]. When the nucleophilic reaction between NH_2_ and O–C=O at the edge of GO occurs in the PPD structure, and nucleophilic substitution reaction occurs with the surface C–O–C of the GO structure, then C–N will be formed [27,33,34].

In order to understand the changes of GO functional groups under different mass ratios of *X* during functional modification, we studied the change of relative strength of different species C with *X*, as shown in Figure 3f. When *X* > 2, the relative strengths of species follow the order C4 > C5 > C3 > C1 > C2. With the increase of *X*, the strength of C1 reduces to minimal at the initial stage, then remains almost unchanged, suggesting that the C–O bond in O–C=O is weak. Therefore, most O–C=O easily react with PPD molecules, causing a rapid decrease of O–C=O strength, while the residual O–C=O remains stable.

The strength of C2 slowly increases at first, then remains unchanged, indicating that the relative content of C=O first increases, then basically remains unchanged. This is because the PPD first reacted with the O–C=O in the GO molecular structure [32], forming some new C=O bonds. Since the reaction was quick, a small amount of residual O–C=O was no longer involved in the reaction, and no new C=O was generated. The strength of C3 decreases rapidly at first, then increases slightly and then decreases slowly, and finally remains unchanged. This is because the covalent reaction between C–O–C and PPD molecules caused the decrease of C–O–C content. However, due to the generation of a small amount of C–OH in the reaction and the reduction of PPD molecules, a small amount of C–OH structure was destroyed. Therefore the species strength reduced, but the C–OH still was not completely eliminated [35].

The strength of C4 increases gradually, which indicates that the relative content of C–C/C=C increases. This is because a large number of oxygen-containing functional groups in the GO structure were destroyed, leading to the restoration of the SP2 structure. Contrary to the expectation, the strength of C5 does not increase with *X*, but displays a change tendency of increasing rapidly first, and then fluctuating around certain value, which indicates that the C–N content in the structure does not increases with the doping amount of PPD. This is probably because the N atoms entered into the graphene oxide network structure, and destroyed the SP2 structure of graphite oxide, which is consistent with the FT-IR results. There are several configuration types of N atoms in the structure. In order to further define the N configuration type in the NRGO-*X* structure, peak-differentiating and imitating of the N1s spectrum was conducted. The detailed discussions are shown below.

**Figure 4:**
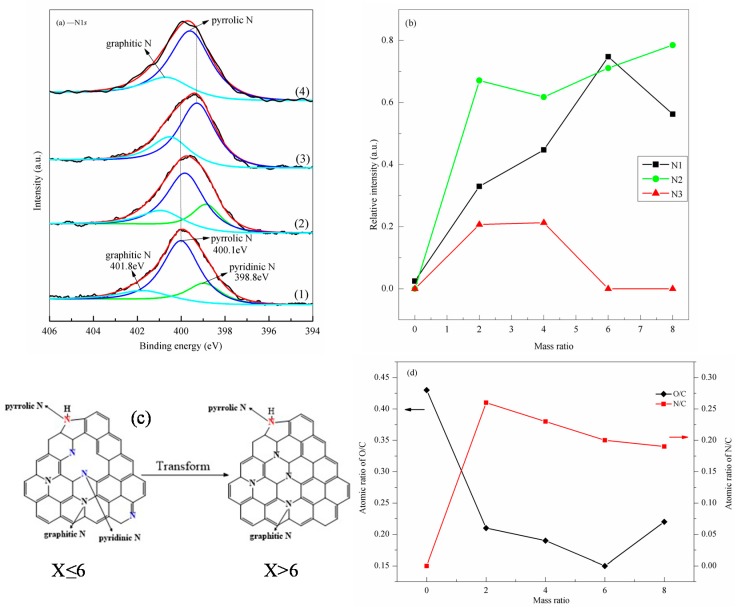
(**a**) N1*s* spectra of NRGO-*X* (*X* = 2, 4, 6, 8), (1) NRGO-2, (2) NRGO-4, (3) NRGO-6, (4) NRGO-8; (**b**) Changes of relative strengths of different N species with the *X*; (**c**) Configuration types of nitrogen atoms in NRGO-X under different ratios of *X*; (**d**) Changes of O/C and N/C with the *X*.

Figure 4a shows that the N atoms of NRGO-2 and NRGO-4 exist in three different forms. The peaks at 401.8 eV, 400.1 eV, and 398.8 eV are ascribed to the graphite oxide skeleton N (labeled as N1), pyrrole N (N2), and pyridine N (N3) [33], respectively. In contrast, the N atoms in NRGO-6 and NRGO-8 exist in two forms, i.e., pyrrole N and graphite oxide skeleton N. At the critical point when the mass ratio *X* is around 6, the N configuration type in NRGO-*X* is transformed.

In addition, for different samples but with the same species N position, peak shift occurs with the increase of *X*. For example, in NRGO-*X*, the binding energy of graphite oxide skeleton N and pyrrole N first shift to the low energy side, then to the high energy side. This is because the electron density of NRGO-*X* was changed and the actions of reduction and nitridation existed in the reaction. When *X* was small, “–CN” and “–NH” groups were formed and increased in amount, making the N1s binding energy decrease. When *X* was large, “–CN” and “–NH” groups were converted into “NO*_X_*” groups, and the N1s binding energy shifted to high energy.

In order to further clarify the changes of different N species types, the change of relative strength of N species was studied, as shown in Figure 4b. It can be seen that with the increase of *X*, the relative strengths of different N species follow the order N2 > N1 > N3. Only around *X* = 6, the relative content of N1 is higher than that of N2, which means the amount of graphite oxide skeleton N introduced to the graphene oxide carbon structure at this point was the most. The N1 strength first increases and then decreases, indicating that graphite oxide skeleton N in graphene oxide carbon network increased first and then decreased. The relative content of graphite oxide skeleton N in NRGO-6 is the highest, which may be because the reaction between PPD and GO molecules was the dominant covalent reaction when *X* ≤ 6.

With the increase of *X*, the amount of graphite oxide skeleton N increases gradually. When *X* > 6, oxygen-containing groups in the structure no longer participate in the covalent reaction, but are gradually reductively eliminated by the excess of PPD molecules. According to the analysis results of Figure 3a, when *X* is large, some of the “N” groups are separated from structure in the form of “NO*_X_*” groups; therefore, the relative content of graphite oxide skeleton N further decreases. The strength of N2 generally increases. Unlike the decreasing strength of N1 at *X* = 6, and the strength of N2 decreases slightly at *X* = 4. The strength of N3 first increases, then remains unchanged, and finally decreases to zero. This is because in the initial reaction stage, the amounts of pyrrole N and pyridine N introduced to the structure both increased (pyrrole N has priority), and more defects were introduced in the structure. However at *X* = 4, part of the N pyrrole structure begins to transform to the graphite oxide skeleton N, and the increasing speed of the relative content of graphite oxide skeleton N from NRGO-2 to NRGO-4 is even faster.

From the above analyses, it was found that GO was reduced and doped by N simultaneously in the reaction. In order to quantitatively study the reduction of GO and N doping level, the changes of O/C and N/C atomic ratio under different values of *X* were calculated according to the sensitivity factor of C, N, O, and the peak intensity, as shown in Figure 4d. It can be seen that the O/C ratio decreases first and then increases, and then reduces from a maximum of 0.43 to 0.15. In contrast, the atomic ratio of N/C can reach 0.26. Although N/C will slightly decrease with the increase of *X*, the strength remains around 0.2, which is still a high level. Therefore, changing *X* is a simple method for preparing NRGO with different reduction and N doping degrees without adding a catalyst.

### 3.2. Layer Structure Changes at Different Ratios of X

**Figure 5:**
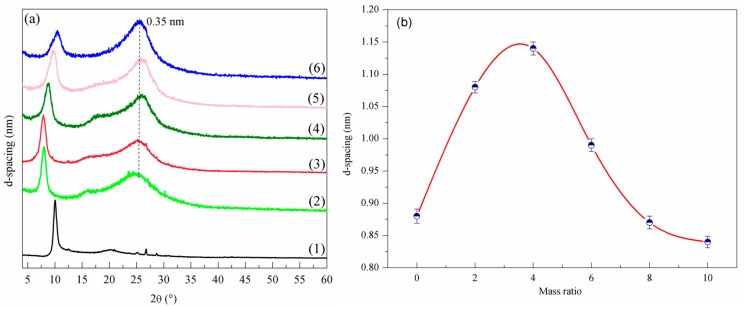
(**a**) XRD patterns of GO and NRGO-*X* (*X* = 2, 4, 6, 8, 10), (**b**) *d*-spacing, (1) GO, (2) NRGO-2, (3) NRGO-4, (4) NRGO-6, (5) NRGO-8, (6) NRGO-10.

Characteristic diffraction peaks 2θ of GO and NRGO-*X*, as well as *d*-spacing, are shown in Figure 5. It can be seen that the layer spacing *D* value of GO is 0.88 nm when 2θ is 10.1°. Meanwhile, the diffraction peaks of the NRGO-*X* series samples are shifted against GO, with corresponding *D* values of 1.08, 1.14, 0.99, 0.87, and 0.84 nm, respectively. It can be seen that with the increase of *X*, the *D* value of NRGO-*X* increases first and then decreases, basically in a parabola distribution. According to the results of the XPS analysis, this is because, with the increase of PPD molecules between the NRGO-*X* layer, the interlayer spacing kept increasing until it reached the maximum value.

From then on, the reduction action of PPD gradually increased, oxygen-containing functional groups in the GO structure were further eliminated, and the layer spacing decreased constantly. However, the C–OH layer in the structure was relatively stable, and could not be completely eliminated. The residual amount of the O–C=O structure was no longer involved in the reaction; therefore, the layer space tended to be constant. In addition, a new peak shoulder appears when the layer spacing *D* value is 0.35 nm, which is due to the irregular stacking of reduced GO sheets after being reduced by PPD.

### 3.3. Conjugate Structure, Electrical Conductivity Change, and Morphology

**Figure 6:**
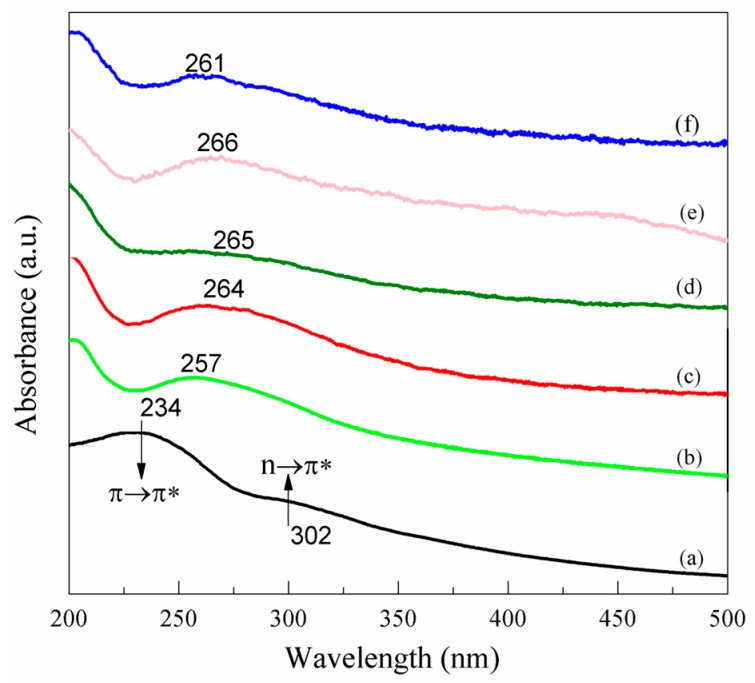
UV-vis spectra of (**a**) GO, (**b**) NRGO-2, (**c**) NRGO-4, (**d**) NRGO-6, (**e**) NRGO-8, (**f**) NRGO-10.

Figure 6 shows the UV-vis spectra of GO and NRGO-*X*. GO exhibited two main characteristic peaks. One characteristic peak occurs when λ_max_ is 234 nm, which is caused by the π → π* transition of the aromatic ring C=C. The other one occurs when λ_max_ is 302 nm, which is ascribed to the n → π* transition of C=O [27]. After the occurrence of the reaction between GO and PPD monomers, the absorption peak of NRGO-2 shifts to 257 nm and the absorption peak increases slightly with the increase of *X*. This is because when –NH_2_ with a lone electron pair connected with a π bond, p-π conjugated effect took place, which increased the range of motion of the electrons, stimulated π → π* transition, and caused the absorption band to move along the long wave direction. This indicates the reduction of GO and the formation of the conjugate structure in NRGO-*X* lamellae. In addition, due to the reaction between PPD and GO containing oxygen functional groups, as well as the strong reduction effect of PPD, the absorption shoulder at 302 nm almost disappears, which is consistent with the results of the XPS analysis.

**Figure 7:**
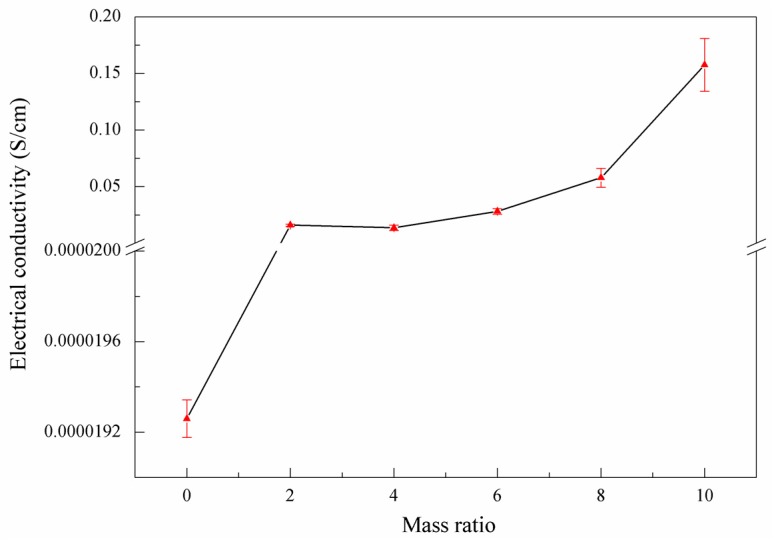
Electrical conductivity of GO (Mass ratio = 0) and NRGO-*X*.

To further analyze what changes the conjugate structure recovery would bring, we measured the resistivity changes, and transformed them into electrical conductivity, as shown in Figure 7. It can be seen that the electrical conductivity of GO is extremely low, while the electrical conductivity of NRGO-*X* increases significantly. The electrical conductivity of NRGO-*X* generally increases with *X*. Combining with the UV-vis results, it was found that such changes are mainly because after the graphite was oxidized, oxygen-containing functional groups and π electrons interacted with each other, and thus the π bond conjugated structure was damaged. Therefore, the electron mobility and electrical conductivity decreased. Through functional modification, the π bond conjugated structure was partially recovered, and the electron mobility increased. In addition, the conjugated structure of the PPD monomers crosslinked with the GO layer, which further expanded the delocalization range of the electron conjugated system of NRGO-*X*.

**Figure 8:**
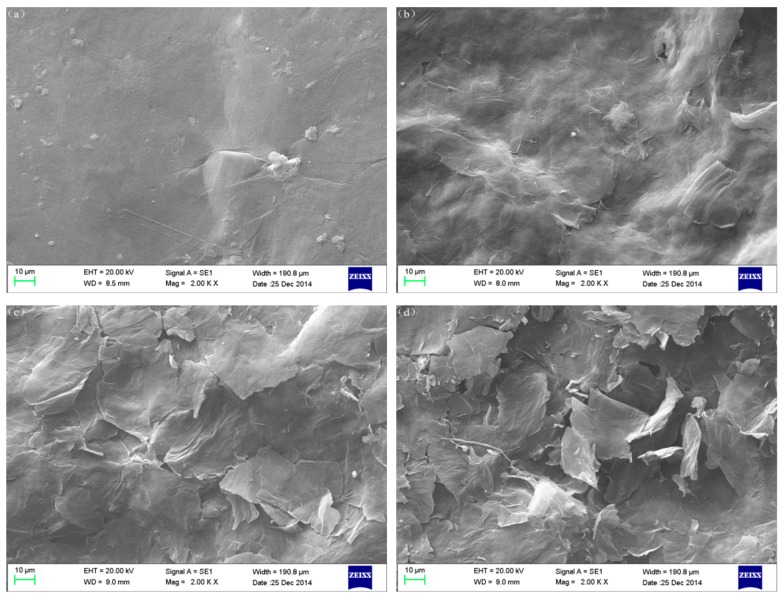
SEM images of samples, (**a**) GO, (**b**) NRGO-2, (**c**) NRGO-4, (**d**) NRGO-6.

For the morphology, the most significant difference between GO and NRGO is surface roughness, as seen from the scanning electron microscope (SEM) images (Figure 8). The surface of GO lamellae is smooth, while NRGO is rougher, and the degree of roughness would increase with increasing PPD. This is mainly due to the rigid structure of PPD monomers, which could inhibit the stacking of sheets and lead to an arrangement of irregular interlayer dislocations. With increasing PPD, this inhibition effect would be enhanced. Meanwhile, due to the elimination of oxygen-containing functional groups, structural defects increased, thus surface roughness increased accordingly.

## 4. Conclusions

(1) PPD was selected as a nitrogen source and modifier. During the reaction, GO can be reduced and doped with N. The reduction of GO as well as the type and extent of N doping can be regulated by changing the mass ratio, *X*. At *X* = 2, the N/C atomic ratio can reach 0.26, while the O/C ratio decreased from 0.43 to 0.15.
(2) When *X* ≤ 2, the changes of relative strengths of different C species were not the same. When *X* > 2, the relative strength of C species followed the order: C–C/C=C > C–N > C–O–C/C–O > O–C=O > C=O. When *X* < 6, there were three types of N atomic configurations in NRGO-*X*, i.e., pyrrole N, pyridine N, and graphite carbon skeleton N. When *X* ≥ 6, there were only two configuration types, that is, pyridine N and graphite carbon skeleton N existing in the NRGO-*X* structure. The relative strength of graphite skeleton N reached the highest value only when *X* = 6. At other mass ratios of *X*, the relative strength followed the order: pyrrole N > graphite skeleton N > pyridine N.
(3) With the increase of *X*, the layer spacing D value of NRGO-*X* first increased and then decreased, generally in a parabolic distribution, while the electrical conductivity increased gradually.
